# Management of impacted maxillary central incisor and abnormality of labial frenulum in children: case report

**DOI:** 10.11604/pamj.2022.42.158.35122

**Published:** 2022-06-28

**Authors:** Tiarisna Hidayatun Nisa, Prawati Nuraini, Sindy Cornelia Nelwan, Seno Pradopo, Soegeng Wahluyo, Betadion, Udijanto Tedjosasongko, Ilvana Adiwirastuti, Barnabas Bonardo

**Affiliations:** 1 Department Pediatric Dentistry, Dentistry Faculty, Universitas Airlangga, Surabaya, Indonesia

**Keywords:** Impacted incisor, surgical exposure, frenectomy, case report

## Abstract

Deposition of the maxillary permanent central incisor is a rare occurrence in dental practice. It is a difficult condition to treat due to its importance to facial esthetics. If complications are to be avoided, early detection of such teeth is important. The present case report of impacted maxillary central incisor encased within an abnormally thickened labial frenulum. A 9-year-old boy, came with his parents to the Pediatric Dentistry Specialist, Dental and Oral Hospital, Airlangga University (UNAIR) with a chief complaint that his left maxillary front teeth did not grow while his right front teeth had grown perfectly. From the anamnesis, the patient had experienced a falling trauma when he was young, which caused the deciduous tooth to fall out. Good general health, no history of allergies, and no medical history of case management. This is a fixed orthodontic treatment with surgical exposure of impacted teeth and frenectomy of labial frenulum. After the crown of the impacted incisor was surgically exposed, eruption ball chain was bonded to traction the incisor. The left maxillary incisor fully erupted and normally to percussion, mobility, and sensitivity testing with good attached gingiva in the next 9 months. Management abnormality of labial frenulum in this case with frenectomy by using electrocautery for minimalized trauma in children. Fixed orthodontic therapy was continued to achieve proper alignment leading to good esthetic and functional rehabilitation. The treatment of an unerupted tooth will depend on its state, position, and presence of enough space in the dental arch to accommodate.

## Introduction

Missing upper incisors can have a substantial impact on dental and facial aesthetics, and are regarded as the least pleasing occlusal feature in US studies [1]. Few studies have reported functional problems associated with missing anterior teeth, although some speech difficulties have been reported, particularly the “s” sound [2]. Because the absence of upper incisors is considered unattractive, it can affect self-esteem and overall social interaction, so it is important to identify and treat the problem early [1]. Usually, once two-thirds of the rooting is complete, the tooth will grow. Impacted teeth do not break through the dental arch in the expected time. Studies have shown that some teeth that do not erupt after the normal eruption time require surgical exposure and orthodontic rearrangement to their normal physiological position in the dental arch [2]. The present case report of impacted maxillary central incisor encased within an abnormally thickened labial frenulum.

## Patient and observation

**Patient information:** a 9-year-old boy, came with his parents to the Pediatric Dentistry Specialist, Dental and Oral Hospital, UNAIR, with a chief complaint that his left maxillary front teeth did not grow while his right front teeth had grown perfectly. From the medical history, the patient had experienced a falling trauma when he was young, which caused the deciduous tooth to fall out. The report showed good general health, no history of allergies, and no medical history of case management.

**Clinical findings:** the clinical assessment indicated an orthognathic facial profile with acceptable facial balance in all dimensions. Except for the left upper central incisor, an intraoral examination indicated the existence of all permanent teeth. The Cephalometric analysis can be seen in Table 1.

**Table 1:** cephalometric analysis

Parameter	Normal	Patient	Analysis
SNA	**83,3 ± 2°**	**82°**	**Normal**
SNB	**81 ± 2°**	**78°**	**Md Hypo**
ANB	**2,5 ± 2°**	**4°**	**Class I**
A-M line	**2 ± 2 mm**	**4 mm**	**Normal**
B-M line	**-1 ± 2 mm**	**2 mm**	**Normal**
Wits	**1 ± 2 mm**	**2 mm**	**Class I**
Go-Gn-Sn	**32 ± 3°**	**30°**	**Normal**
FMA	**26 ± 3°**	**28°**	**Normal**
I - SN	**109 ± 6°**	**96°**	**Normal**
I - MP	**96 ± 5°**	**92°**	**Normal**
I - I	**125 ± 5°**	**138°**	**Retroclination**
Naso labial angle	**93 ± 3°**	**95°**	**Normal**
Lower lip - E line	**1 ± 1 mm**	**2 mm**	**Normal**

**Figure 1:**
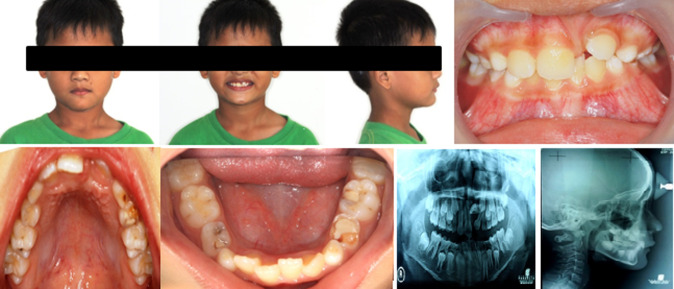
pre-treatment profile, intra-oral, and radiograph view

**Diagnostic assessment:** to have a solid picture of the location and morphology of the unerupted right permanent central incisor in the maxilla, cephalometric and panoramic (orthopantomogram or OPG) radiographs were acquired. At the mucogingival junction, a tooth bulged in the labial sulcus. Figure 1 showed the orthognathic facial profile.

**Diagnosis:** it was located quite high up in the alveolar bone, with a thick layer of soft tissue covering the crown vertically. The crown of the erupted permanent left central incisor was 8 mm wide at its widest point. The maxillary space available for an unerupted right permanent central incisor was 8 mm.

**Figure 2:**
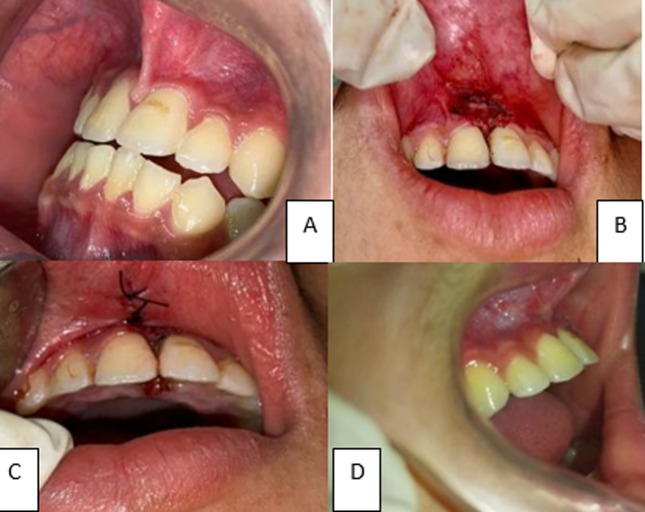
(A) pre-operation, (B) frenectomy with electrocautery, (C) suturing, (D) removal of the surgical suture after 1 week

**Dental intervention:** management of abnormality of labial frenulum in this case is using frenectomy with electrocautery for minimalized trauma in children. Frenectomy is a surgical treatment that can be done with a scalpel, electrosurgery, or laser [3,4]. In the electrocautery technique, the spot was anesthetized with articaine 4% with 1: 100.000 epinephrine. The hemostat was used to raise the tissue, keep it firm, and introduce it into the vestibule's depth. The electrosurgery unit ART-E1 (Bonart Co. Ltd, Taipei country, Taiwan) was utilized. The cutting electrode was programmed with 4 RF/2MHz, a power supply of 23010 percent 50/60 Hz, and a current of 0.9A 210 VA. The output power was fixed at 38 watts RMS + 5%. (Compared with 18 watts from a standard Valley Lab Electrocautery). The working frequency was reduced by 5% to 1.5 MHz. The electrode was used to make two incisions. While the electrocautery was being used, continuous saline irrigation was administered. The hemostat was next used to release the triangular tissue of the labial frenulum. The excision region was coated with alveolar gel before suturing. It was also sutured using 3.0 silk for three stitches. The patient was recalled after 1 week for suture removal and after 1 month for follow-up as seen in Figure 2.

**Figure 3:**
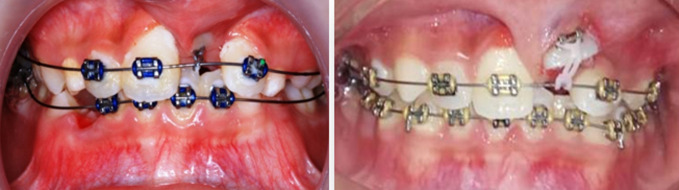
after surgical exposure, the eruption ball was bonded to impacted incisor

It was decided to perform surgical exposure of the impacted tooth, followed by bonding a bracket to the tooth labial surface and bringing it down to its natural position. Mini-Roth brackets were bonded to the permanent right upper central and lateral incisors, while molar tapes were used for anchorage. After the crown of the affected incisor was exposed surgically, a bead-sprayed chain bracket was bonded on the exposed incisor, and high-grade 0.016 NiTi was used to align the right central incisor which can be seen in Figure 3.

**Figure 4:**
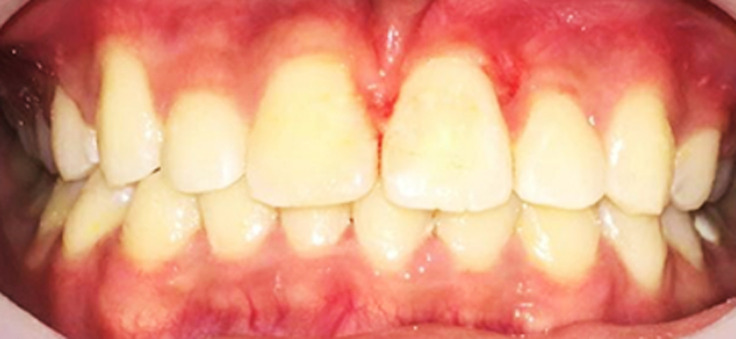
intra-oral after-braces were debonding

**Follow-up and outcome of interventions:** the patient´s clinical crown length was normal, and the gingival contour and width were adequate. The patients were satisfied with the cosmetic results as seen in the following Figure 4. The maxillary right incisors survived well, reacted well to tapping, mobility, and sensitivity tests, and had a modest connecting gingival width at the 9-month follow-up. After all treatment objectives have been met, including an optimal overjet and overbite with a well-interdigitated class I molar relationship, the patient data did not change noticeably. The central incisors' gingival contour is acceptable. Instead of the lateral incisor, the upper right canine was modified. To avoid traumatic occlusion and provide adequate canine-protected occlusion, occlusal modification on the upper right first premolar was done. The maxillary and mandibular teeth were stabilized using Hawley retainers once the fixed device was removed. After wearing the retainers all day for 6 months, they were only worn at night for another 1.5 years.

**Patient perspective:** the patient feels satisfied with the treatment result.

**Patient consent:** the informed consent is provided by the Dental and Oral Hospital of Airlangga University and given to the patient. The patient agreed to the informed consent, including his picture posted.

## Discussion

An abnormality in the eruption of anterior teeth can have an impact on face esthetics and generate psychological issues. As a therapy option, several strategies have been created. If the affected tooth is removed, alveolar bone loss is expected. Lin *et al*. [5] stated that the loss of the aveolar bone due to resorption is almost unavoidable, and it usually occurs in the first six months after extraction. Following the healing phase, the alveolar ridge becomes thinner and weak; with these disadvantages in mind, the aims of orthodontic treatment become assisting natural tooth eruption and maintaining natural look. As a result, surgical exposure and orthodontic treatment options for such impacted teeth are accepted. Several studies have found that an impacted tooth may be realigned in the dental arch. However, some circumstances may have an effect on whether an impacted tooth can be successfully aligned: (1) the impacted tooth's position and orientation, (2) the degree of root completeness, (3) the degree of dilacerations, and (4) the presence of space for the affected tooth [2, 6].

To prevent unnecessary complications in aligning the tooth in the arch, orthodontic and surgical treatments should not be postponed [7]. Several surgical procedures for exposing impacted teeth before orthodontic tooth movement have been described [8]. Labial impacted teeth have two most common surgical exposure techniques, including: (1) exposing the entire labial aspect of the anatomic crown with total excision of all keratinized tissue (the window approach) and (2) exposing only 4-5 mm of the most superficial portion of the labial aspect of the cusp tip while retaining 2-3 mm of keratinized tissues [9, 10]. The available room for tooth alignment was sufficient in this situation, and the tooth was brought into the correct anatomical position in the dental arch. It has been proposed and demonstrated that the “window” technique results in statistically significant loss of attachment, recession, and gingival irritation on the maxillary incisor after surgical exposure [11]. As a result, either keratinized gingiva or an apical flap must be maintained. This method seeks to achieve keratinized gingiva all the way around the emerging tooth. It is critical for a tooth to emerge via connected gingival rather than alveolar mucosa [11]. If the impacted tooth is diagnosed with its root fully grown or if it is in an unfavorable position, a combination of surgical and orthodontic therapy is required.

## Conclusion

The treatment of an unerupted tooth will be determined by its condition, location, and the existence of sufficient space in the dental arch to accommodate it. When the impacted tooth is diagnosed with its root completely developed or in an unfavorable position, surgical and orthodontic therapy are necessary. The use of electrocautery improved patient comfort by reducing intra-operative bleeding, discomfort, edema, epithelization, infection, and operating time. For surgical intraoral procedures in children, electrocautery is indicated. Multidisciplinary collaboration with a full diagnostic and realistic treatment plan should be done for the optimal management of impacted teeth to obtain optimum cosmetic and functional results. In the meanwhile, periodontal health factors should be taken into account to achieve desired results.
